# Outcomes of a Digitally Delivered Low-Carbohydrate Type 2 Diabetes Self-Management Program: 1-Year Results of a Single-Arm Longitudinal Study

**DOI:** 10.2196/diabetes.9333

**Published:** 2018-08-03

**Authors:** Laura R Saslow, Charlotte Summers, James E Aikens, David J Unwin

**Affiliations:** 1 Department of Health Behavior and Biological Sciences School of Nursing University of Michigan Ann Arbor, MI United States; 2 Diabetes.co.uk Coventry United Kingdom; 3 Department of Family Medicine University of Michigan Medical School University of Michigan Ann Arbor, MI United States; 4 Principal in General Practice, The Norwood Surgery Southport United Kingdom

**Keywords:** eHealth, diet, weight loss, type 2 diabetes mellitus

## Abstract

**Background:**

Type 2 diabetes mellitus has serious health consequences, including blindness, amputation, stroke, and dementia, and its annual global costs are more than US $800 billion. Although typically considered a progressive, nonreversible disease, some researchers and clinicians now argue that type 2 diabetes may be effectively treated with a carbohydrate-reduced diet.

**Objective:**

Our objective was to evaluate the 1-year outcomes of the digitally delivered Low-Carb Program, a nutritionally focused, 10-session educational intervention for glycemic control and weight loss for adults with type 2 diabetes. The program reinforces carbohydrate restriction using behavioral techniques including goal setting, peer support, and behavioral self-monitoring.

**Methods:**

The study used a quasi-experimental research design comprised of an open-label, single-arm, pre-post intervention using a sample of convenience. From adults with type 2 diabetes who had joined the program and had a complete baseline dataset, we randomly selected participants to be followed for 1 year (N=1000; mean age 56.1, SD 15.7 years; 59.30% (593/1000) women; mean glycated hemoglobin A_1c_ (HbA_1c_) 7.8%, SD 2.1%; mean body weight 89.6 kg, SD 23.1 kg; taking mean 1.2, SD 1.01 diabetes medications).

**Results:**

Of the 1000 study participants, 708 (70.80%) individuals reported outcomes at 12 months, 672 (67.20%) completed at least 40% of the lessons, and 528 (52.80%) completed all lessons of the program. Of the 743 participants with a starting HbA_1c_ at or above the type 2 diabetes threshold of 6.5%, 195 (26.2%) reduced their HbA_1c_ to below the threshold while taking no glucose-lowering medications or just metformin. Of the participants who were taking at least one hypoglycemic medication at baseline, 40.4% (289/714) reduced one or more of these medications. Almost half (46.40%, 464/1000) of all participants lost at least 5% of their body weight. Overall, glycemic control and weight loss improved, especially for participants who completed all 10 modules of the program. For example, participants with elevated baseline HbA_1c_ (≥7.5%) who engaged with all 10 weekly modules reduced their HbA_1c_ from 9.2% to 7.1% (*P*<.001) and lost an average of 6.9% of their body weight (*P*<.001).

**Conclusions:**

Especially for participants who fully engage, an online program that teaches a carbohydrate-reduced diet to adults with type 2 diabetes can be effective for glycemic control, weight loss, and reducing hypoglycemic medications.

## Introduction

Type 2 diabetes mellitus is prevalent, costly, and a potentially progressive disease with serious health consequences including blindness, amputation, stroke, dementia, and premature death [1]. Globally, one in 11 people, or 422 million adults, have diabetes (with most of those cases being type 2 diabetes) [2]. It is the most expensive disease in the United States [3], and its annual global costs are more than US $800 billion [4]. In community settings, type 2 diabetes is rarely reversed. For example, a study that followed more than 100,000 patients with type 2 diabetes over 7 years found that less than 1% of patients experienced complete remission [5].

Although typically considered a progressive, nonreversible disease, some researchers and clinicians now argue that type 2 diabetes may be effectively treated with a carbohydrate-reduced diet, which could improve type 2 diabetes management and potentially even lead to remission [6]. Indeed, previous research with carbohydrate-reduced diets for type 2 diabetes do show improved outcomes (eg, glycemic control, weight loss, and reductions in the use of hypoglycemic medications) for both very low-carbohydrate diets (approximately 20% or fewer of total dietary calories derived from carbohydrates) [7-9] or lower carbohydrate diets (approximately 40% or fewer of total dietary calories derived from carbohydrates) [10,11].

Although dietary interventions have historically been in-person, online programs can be just as effective for some participants, as suggested by research that has examined diet and lifestyle interventions in adults with prediabetes [12]. Therefore, it is perhaps not surprising that the beneficial results of carbohydrate-reduced diets for people with type 2 diabetes (glycemic control, weight loss, and reductions in the use of hypoglycemic medications) have been replicated using online programs [13,14]. Notably, both previous trials of a very low-carbohydrate diet online for adults with type 2 diabetes included the use of a coach. However, previous research on weight loss (including some people with type 2 diabetes), have shown some success with a completely automated online weight loss program, with approximately 50% of participants losing at least 5% of their body weight by 6 months [15,16].

In this naturalistic pilot study, our objective was to evaluate the 1-year outcomes of the Low-Carb Program, a digitally delivered, nutrition-focused, structured lifestyle intervention with 10 weekly sessions for glycemic control, hypoglycemic medication use, and weight loss for adults with type 2 diabetes. We hypothesized that this program would lead to improvements compared to baseline: better glycemic control (as measured by glycated hemoglobin A_1c_ or HbA_1c_), weight loss, and reductions in hypoglycemic medication use. Our goal was to explore whether the program might be an effective option for increasing access to diabetes management solutions and help halt the prevalent, costly, and dangerous type 2 diabetes epidemic.

## Methods

### Research Design

We used a quasi-experimental research design comprised of a single-arm pre-post intervention. Participants were not paid for their participation, but because the program was free, they took part in the program at no cost. The University of Michigan Institutional Review Board (IRB) ruled that analyses of these previously collected and de-identified data were not subject to IRB regulation.

### Participants

**Figure 1:**
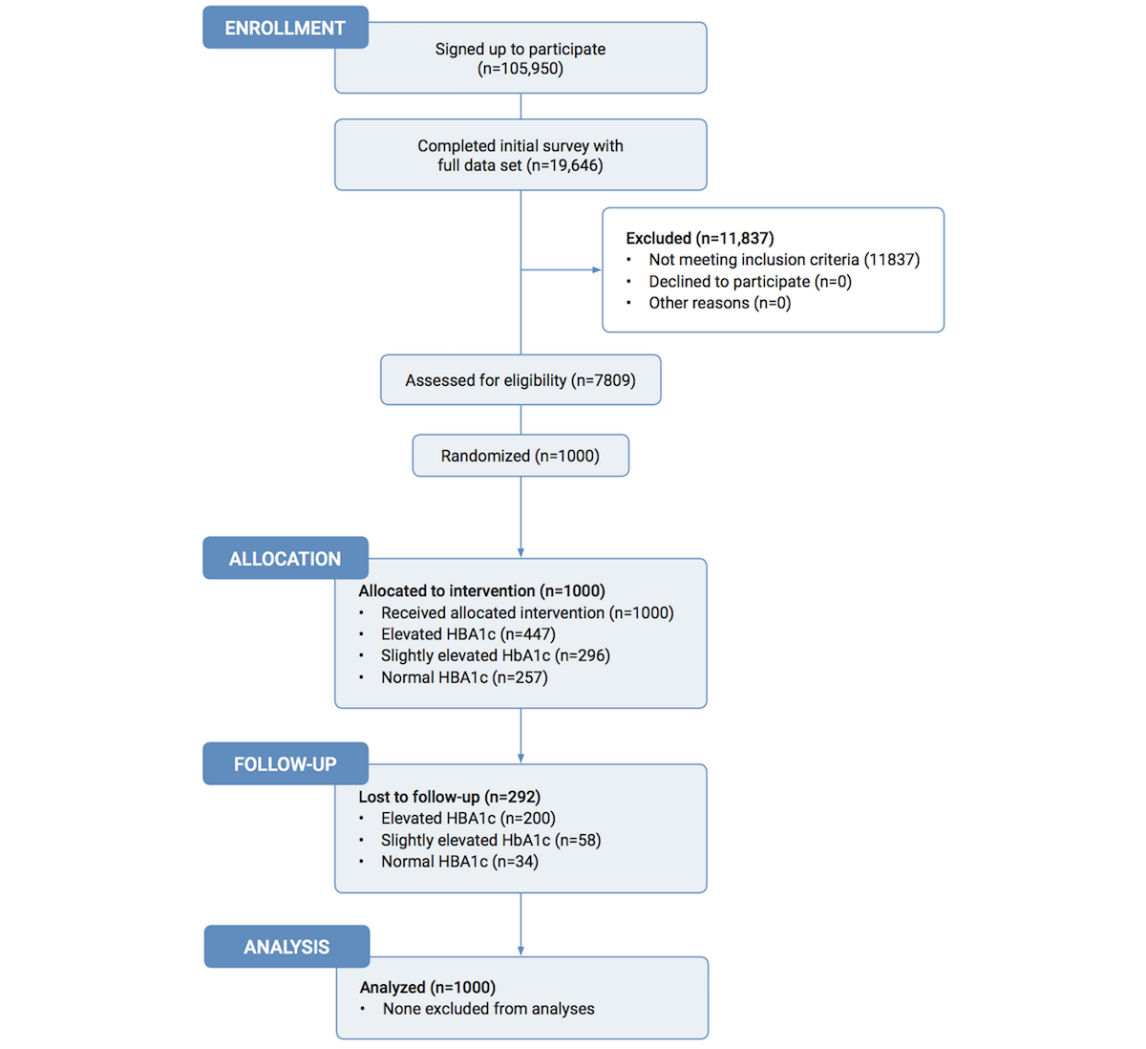
Study participant ﬂowchart for the study.

We recruited participants to this trial in three phases. The first phase recruited a sample of convenience following the launch of the Low-Carb Program (November 14, 2015-November 14, 2016), whereby 105,950 adults with type 2 diabetes between the ages of 18 and 99 years signed up to participate in the program. Participants could live anywhere in the world. To have a broad applicability to a nonclinical trial setting, the only de facto exclusion criterion was the inability to understand English. Second, upon sign-up, the program prompted individuals to complete an initial baseline survey; 19,646 of 105,950 (18.54%) did so. Of those, 7809 people had complete baseline datasets including weight, a recent HbA_1c_ result (taken within 4 months), and medication use. Third, we used GraphPad Random Generator Software to randomly select a subset of 1000 participants to be followed for 12 months, thus enabling us to select participants for no other reason than that they were randomly selected by the software. Therefore, we did not include all the 7809 patients to follow over a year, but instead followed a random subsample of 1000 (see Figure 1).

### The Low-Carb Program

The Low-Carb Program is a completely automated, structured 10-week health intervention for adults with type 2 diabetes. Participants are given access to nutrition-focused modules, with a new module available each week over the course of 10 weeks. The modules are designed to help participants gradually reduce their total carbohydrate intake to less than 130 grams per day to meet their self-selected goals. The program encourages participants to make behavior changes based on “action points” or behavior change goals at the end of each module. These goals are supported with resources that are available to download, including information sheets, recipes, and suggested food substitution ideas. The Low-Carb Program online platform also includes digital tools for submitting self-monitoring data on a number of different variables including blood glucose levels, blood pressure, mood, sleep, food intake, and body weight. Weekly automated feedback is provided to users based on their use of the program through email notifications, and participants are notified when the next week’s module has been opened. Lessons are taught through videos, written content, or podcasts of varying lengths (approximately 3 to 12 minutes long).

The first 2 weeks of the program contain an explanation of the physiology of type 2 diabetes and the role of diet, including a description of how a low-carbohydrate diet can help manage postprandial blood glucose levels and weight. The subsequent week’s modules explore strategies to reduce dietary sources of sugar, in particular, high-starch foods, such as bread, pasta, and rice. Participants are encouraged to make portion control and carbohydrate restriction decisions based on visual plate representations. In place of carbohydrate-rich foods, an increased intake of green vegetables, low-glycemic index fruits (eg, blueberries, strawberries, and raspberries) and fats (eg, from olive oil, butter, eggs, nuts, and full-fat dairy) are advocated. The program stresses the importance of regular contact with the participants’ health care providers for adjustments in medications in weeks 1, 2, and 10. After the 10 weeks of modules have been opened, participants continue to have access to the education content as well as the ability to continue to track their health (glycemic control, weight) and access support from the discussion board. See Table 1 for a list of the weekly topics.

Much of the content of the Low-Carb Program is based on an in-person, nurse- and physician-led, low-carbohydrate training program conducted in a primary health care setting [17]. For example, the dietary recommendations reflect an understanding of the glycemic index, a relative ranking of carbohydrates in foods according to how they affect blood glucose levels. A meal of pure glucose (the index food) has a score of 100, boiled potatoes are scored at 96, cornflakes at 93, and brown bread at 74, all of which are higher than table sugar at 63 [18]. This kind of information helps participants understand that both sugary and starchy foods increase blood glucose, and it also explains why the UK’s National Institute for Health and Care Excellence advises physicians to “encourage high-fiber, low-glycemic index sources of carbohydrate in the diet” for type 2 diabetes [19]. Based on this, the program suggests a reduction in all sugary foods and replacing starchy foods, such as potato or rice, with green leafy vegetables, healthy fats, and some protein.

**Table 1:** Weekly topics of the Low-Carb Program.

Week	Title	Objective
1	Welcome to the Low-Carb Program	Safety notes and alerts to medications that require health team’s assistance; initiate conversation with health care providers prior to making any dietary adaptations; benefits of a reduced carbohydrate diet for people with type 2 diabetes
2	Type 2 diabetes and diet	Factors that affect blood glucose levels; encouragement to engage with their health care providers
3	Controlling portion sizes	Visual methods of interpreting portion size
4	Processed versus unprocessed foods	Identifying and eliminating refined and processed food
5	Healthy and unhealthy fats	Discussion of fat types and making appropriate choices depending on goals
6	Vegetables	The carbohydrate content of vegetables; cooking methods
7	Sugar and starch	Reviewing the amount of sugar and starch in fruit and vegetables
8	Snacks, desserts, and drinks	Examining low-carb snack, dessert, and drink options
9	Alcohol, eating away from home	Alcohol; options for eating away from home
10	Practical ways of reducing carbohydrate intake further	Practical tips for reducing carbohydrate intake further; safety information—highlighting medications that require assistance from their physicians and how to involve their physician and wider health care team

The content and strategies used in the program build off prior research and theory. For example, evidence suggests that goal setting can act as an effective behavior change strategy used to improve adherence to lifestyle intervention programs in obesity management programs [20]. Therefore, the program encourages participants to select a goal at the beginning of the program (eg, to lose weight, reduce medication dependency, or make healthier choices for their whole family). Participants are also prompted to consider how their health would benefit from attaining their goal. Throughout the program, participants are periodically prompted to consider how close they are to attaining their goal.

The program further reinforces behavior change through integrated tracking whereby program users are encouraged to track their health data including mood, food intake, blood glucose levels, weight, sleep, and HbA_1c_. According to the Control Theory of behavior change, monitoring goal progress—that is, evaluating one’s ongoing performance relative to the standard—and responding accordingly is critical to goal attainment [21]. Recent findings suggest that program interventions that elevate the frequency of progress monitoring are likely to induce behavior change [22].

In addition, prior studies demonstrate that peer support may improve blood glucose control [23,24], peer-based support may be as effective for weight loss as coach-based support [25], and that online discussion boards can be supportive for weight loss [26]. Therefore, the program encourages social support by matching new participants of the program to a “buddy,” a previous graduate of the program, based on similar demographics including age, gender, and their self-selected goal. Participants are encouraged to interact with that buddy and peers on the program’s moderated online discussion board.

### Measures

At baseline, an online survey asked participants to report on their type of diabetes, year of diagnosis, their most recent HbA_1c_ test result and date, current medications (medication name, dose, and regimen), age, gender, socioeconomic status (based on household income), and presence of comorbid chronic illnesses. At 12 months, participants were again asked to report on their current HbA_1c_, weight, and medications.

### Statistical Analyses

Analyses were performed using the SPSS version 21.0 (SPSS Inc, Chicago, IL, USA). We examined the difference in characteristics from baseline to 12-month follow-up using paired *t* tests. The primary outcome was change in HbA_1c_ and body weight (kg, percent of initial body weight). The secondary outcome was change in need for diabetes medication. We stratified our cohort into three groups according to baseline glycemic control as defined by baseline HbA_1c_: (1) elevated baseline HbA_1c_ greater than or equal to 7.5%, (2) slightly elevated baseline HbA_1c_ 6.5% to 7.4%, or (3) normal baseline HbA_1c_ less than 6.5%. Outcomes were also analyzed within strata based on participant’s Low-Carb Program completion (ie, completers: engaged with all 10 of the Low-Carb Program weekly modules; n=528), partial completers (engaged with 4-9 modules; n=144), or noncompleters (engaged with ≤3 modules; n=328).

Some of our results took into account the entire sample, regardless of follow-up information or lesson completion. For participants who did not report their outcomes at 12 months, we followed the highly conservative approach of assuming that they did not improve at all (last observation carried forward), by imputing their baseline values as their outcome values. For example, participants who did not comply with reporting a 12-month outcome were treated as having no change in the outcome variable, and thus were not counted as having any HbA_1c_ or weight improvement.

## Results

### Participant Characteristics at Baseline

At baseline, mean HbA_1c_ was 7.8% (SD 2.1%), mean weight was 89.6 kg (SD 23.1), and mean age was 56.1 years (SD 15.7) years. More than half of participants were female (59.3%, 593/1000), 90.4% (904/1000) were white, all were from the United Kingdom, and more than one-third had comorbid hypertension (39.7%, 397/1000) or hypercholesterolemia (35.0%, 350/1000). At baseline, participants were taking a mean of 1.21 (SD 1.01) hypoglycemic medications. See Table 2 for details.

### Retention

Of the 1000 baseline participants, 708 (70.80%) reported outcomes at 12 months, 528 (52.80%) completed all lessons, and 672 (67.20%) completed at least 40% of the lessons. For the remaining 292 people lost to follow-up, the last recorded data point was carried forward. Of 447 people with elevated HbA_1c_ (≥7.5%) at baseline, 247 (55.3%) reported outcomes at 12 months and 191 (42.7%) completed all lessons. Of 296 people with slightly elevated HbA_1c_ (6.5%-7.5%) at baseline, 238 (80.4%) had outcomes at 12 months and 182 (61.4%) completed all lessons. Of 257 people with a normal baseline HbA_1c_ level (HbA_1c_ <6.5%) who began the study, 223 (86.8%) had outcomes at 12 months and 155 (60.3%) completed all lessons (see Figure 1 for the participant flowchart of the study).

**Table 2:** Participant characteristics at baseline.

Characteristic		Pooled (N=1000)	Baseline HbA_1c_ level^a^		
			Elevated (n=447)	Slightly elevated (n=296)	Normal (n=257)
Age (years), mean (SD)		56.1 (15.7)	54.8 (14.6)	56.7 (16.9)	57.9 (15.8)
HbA_1c_ (%), mean (SD)		7.8 (2.1)	9.6 (1.8)	6.90 (0.3)	5.68 (0.7)
Weight (kg), mean (SD)		89.6 (23.1)	92.9 (24.0)	88.2 (22.4)	85.7 (21.8)
**Gender, n (%)**					
	Male	401 (40.1)	175 (39.1)	124 (41.9)	102 (39.7)
	Female	593 (59.3)	271 (60.6)	171 (57.8)	151 (58.8)
	Intersex	6 (0.6)	1 (0.2)	1 (0.3)	4 (1.6)
**Ethnicity, n (%)**					
	White	904 (90.4)	409 (91.5)	259 (87.5)	236 (91.8)
	Indian/Pakistani	36 (3.6)	12 (2.7)	16 (5.4)	8 (3.1)
	Mixed/Multiple ethnic groups	16 (1.6)	6 (1.3)	8 (2.7)	2 (0.8)
	Chinese/Japanese/Other East Asian	8 (0.8)	3 (0.7)	4 (1.4)	1 (0.4)
	Black/African/Caribbean	21 (2.1)	10 (2.2)	5 (1.7)	6 (2.3)
	Unknown	15 (1.5)	7 (1.6)	4 (1.4)	4 (1.6)
**Employment, n (%)**					
	Full-time employment	315 (31.5)	171 (38.3)	88 (29.7)	56 (21.8)
	Part-time employment	135 (13.5)	61 (13.6)	37 (12.5)	37 (14.4)
	Retired	480 (48.0)	179 (40.0)	154 (52.0)	147 (57.2)
	Student	7 (0.7)	3 (7.4)	2 (0.7)	2 (0.8)
	Unemployment	63 (6.3)	33 (0.7)	15 (5.1)	15 (5.8)
**Comorbidities, n (%)**					
	Hypertension	397 (39.7)	184 (41.2)	109 (36.8)	104 (40.5)
	High cholesterol	350 (35.0)	149 (33.3)	105 (35.5)	96 (37.4)
**Medications in current use, n (%)**					
	Insulin	157 (15.7)	102 (22.8)	35 (11.8)	20 (7.8)
	Metformin	596 (59.6)	301 (67.3)	165 (55.7)	130 (50.6)
	Other	452 (45.2)	305 (68.2)	90 (30.4)	57 (22.2)

^a^Elevated: baseline HbA_1c_ ≥7.5%; slightly elevated: baseline HbA_1c_ 6.5%-7.4%; normal: baseline HbA_1c_ <6.5%.

### Changes in Glycemic Control

**Figure 2:**
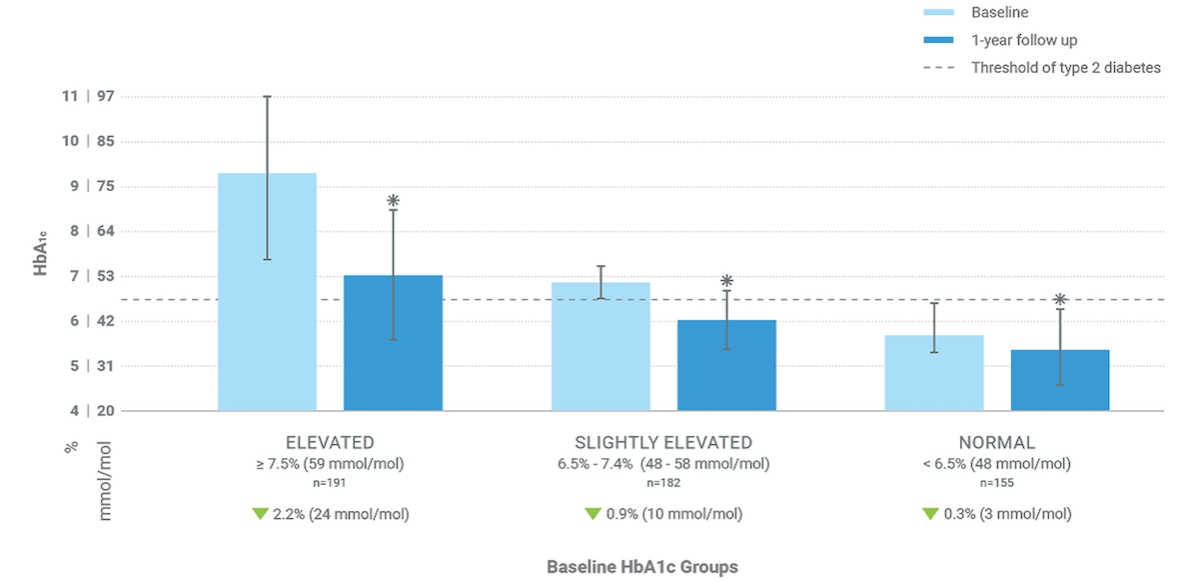
Mean glycated hemoglobin A_1c_ (HbA_1c_) levels at baseline and 1-year follow-up for participants who completed the program (engaged with all 10 weekly Low Carb Program modules). Error bars represent the SD; * represents significant difference from baseline.

Considering all participants pooled across baseline HbA_1c_, those who completed the Low-Carb Program showed a statistically significant change in HbA_1c_ of –1.17% (SD 1.43; *t*_527_=18.724, *P*<.001). Partial completers showed a statistically significant change in HbA_1c_ of –0.6% (SD 1.69; *t*_143_=4.276, *P*<.001) and noncompleters showed a nonsignificant HbA_1c_ change of only –0.16% (SD 1.13; *t*_328_=2.54, *P*=.01). Results stratified by baseline HbA_1c_ are presented in Table 3, and results for just Low-Carb Program completers are presented in Figure 2.

### Body Weight

**Figure 3:**
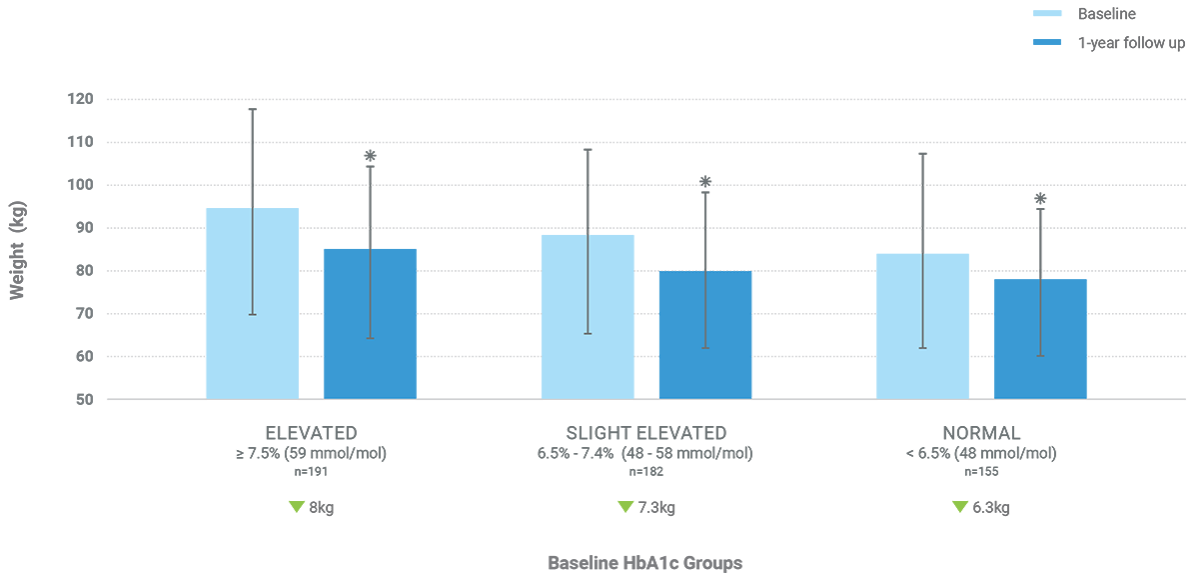
Mean weight at baseline and 1-year follow-up for participants who completed the program (engaged with all 10 weekly Low Carb Program modules). Error bars represent the SD; * represents significant difference from baseline.

Considering all baseline HbA_1c_ groups combined, Low-Carb Program completers (n=528) showed a significant reduction in weight, with a mean body weight change of –7.45 kg (SD 12.63) or –7.0% (SD 12.81%; *t*_527_=13.551, *P*<.001). Partial completers (n=144) showed a reduction in weight, with a mean body weight change of –2.13 kg (SD 16.40) or –1.1% (SD 25.42%); however, this weight change was not statistically significant (*t*_143_=1.563, *P*=.12). Noncompleters (n=328) did not have a statistically significant change in weight, with mean change of –0.35 kg (SD 10.13) or 0.7% (SD 13.41%; *t*_327_=0.625, *P*=.53). Results, stratified by baseline HbA_1c_, are presented in Table 4, and results for just Low-Carb Program completers are presented in Figure 3.

### Hypoglycemic Medications

The majority of participants (714/1000, 71.40%) were prescribed at least one hypoglycemic medication at baseline. At 1 year, of those originally prescribed medications, 289/714 (40.4%) individuals were able to stop one or more hypoglycemic medications. Of the 743 participants who started with an HbA_1c_, equal to or above the type 2 diabetes threshold of 6.5%, 195 (26.2%) reduced their HbA_1c_ to below the threshold while taking no glucose-lowering medications or just metformin.

**Figure 4:**
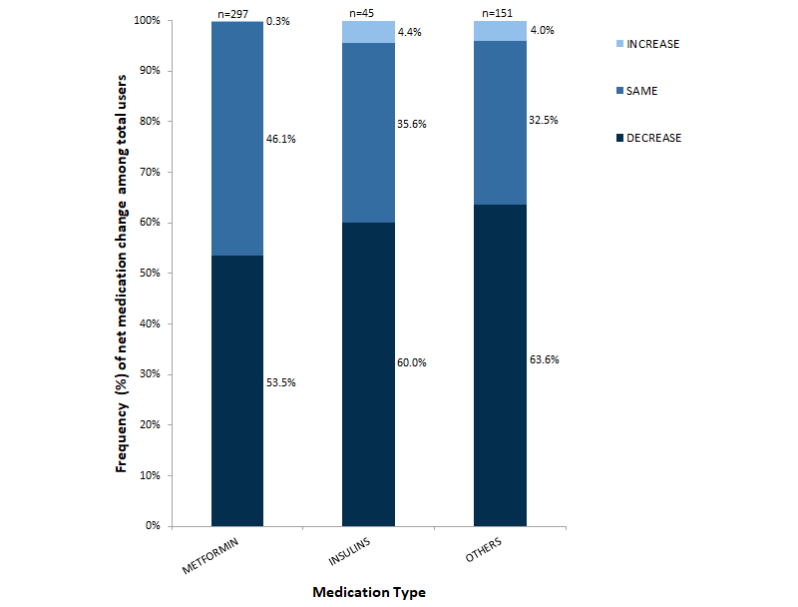
Frequency of change in the number of medications taken for all completers. Bars represent total users of each drug type with the type of change (increase, no change, or elimination) stacked within the bar and the relative frequency noted next to each section. The total number of users of each medication type is noted at the top of each bar.

For participants who completed the program, the proportion prescribed hypoglycemic medications changed significantly between baseline and follow-up for metformin (χ^2^_24_=146.5, *P*<.05) and other hypoglycemic medications (all hypoglycemic medications other than metformin and insulin: χ^2^_24_=73.8, *P*<.05). However, there was no significant change in being prescribed insulin (χ^2^_24_=34.1, *P*=.08; see Figure 4).

**Table 3:** Change in HbA_1c_ from baseline to 1-year follow-up by intervention completion.

Baseline HbA_1c_ group		Baseline HbA_1c_ (%), mean (SD)	1-year HbA_1c_ (%) mean (SD)	HbA_1c_ change (%), mean (SD)	*P* value
**Pooled (all participants)**					
	All participants (N=1000)	7.78 (2.10)	7.03 (2.04)	–0.76 (1.46)	<.001
	Completers (N=528)	7.40 (1.81)	6.23 (1.19)	–1.17 (1.43)	<.001
	Partial completers (N=144)	7.00 (1.72)	6.40 (1.44)	–0.60 (1.69)	<.001
	Noncompleters (N=328)	8.75 (2.33)	8.59 (2.43)	–0.16 (1.13)	.01
**Elevated (HbA_1c_≥7.5%)**					
	All participants (n=447)	9.58 (1.80)	8.36 (2.22)	–1.22 (1.75)	<.001
	Completers (N=191)	9.23 (1.71)	7.06 (1.35)	–2.16 (1.76)	<.001
	Partial completers (N=47)	8.88 (1.37)	7.26 (1.67)	–1.62 (1.97)	<.001
	Noncompleters (N=209)	10.06 (1.84)	9.79 (2.12)	–0.28 (1.06)	<.001
**Slightly elevated (HbA_1c_ 6.5-7.4%)**					
	All participants (N=296)	6.90 (0.28)	6.22 (0.90)	–0.68 (0.89)	<.001
	Completers (N=182)	6.88 (0.27)	6.01 (0.69)	–0.87 (0.68)	<.001
	Partial completers (N=42)	6.92 (0.31)	6.23 (0.86)	–0.69 (0.87)	<.001
	Noncompleters (N=72)	6.93 (0.27)	6.74 (1.18)	–0.19 (1.16)	.16
**Normal (HbA_1c_<6.5%)**					
	All participants (N=257)	5.68 (0.68)	5.65 (0.95)	–0.03 (1.06)	.64
	Completers (N=155)	5.77 (0.61)	5.47 (0.75)	–0.30 (0.75)	<.001
	Partial completers (N=55)	5.45 (0.80)	5.79 (1.22)	0.33 (1.36)	.07
	Noncompleters (N=47)	5.66 (0.69)	6.08 (1.07)	0.42 (1.24)	.02

**Table 4:** Change in participant body weight from baseline to 1-year follow-up for people with elevated or slightly elevated baseline HbA_1c_ by intervention completion amount.

Baseline HbA_1c_ group		Baseline weight (kg), mean (SD)		1-year weight (kg), mean (SD)		1-year percent weight change, mean (SD)		1-year weight change (kg), mean (SD)		*P* value	
**Pooled (all participants)**											
	All participants (N=1000)		89.63 (23.13)		85.28 (20.73)		–3.31 (15.93)		–4.35 (12.93)		<.001
	Completers (n=528)		88.88 (22.16)		81.43 (17.98)		–6.97 (12.83)		–7.45 (12.63)		<.001
	Partial completers (n=144)		87.77 (22.91)		85.64 (19.02)		1.09 (25.51)		–2.13 (16.39)		.12
	Noncompleters (n=328)		91.66 (24.63)		91.31 (23.93)		0.65 (13.41)		–0.35 (10.13)		.53
**Elevated (HbA_1c_≥7.5%)**											
	All participants (N=447)		92.88 (23.96)		89.46 (22.24)		–2.39 (14.70)		–3.42 (12.32)		<.001
	Completers (n=191)		92.98 (23.62)		84.96 (18.85)		–6.94 (13.90)		–8.01 (13.83)		<.001
	Partial completers (n=47)		90.49 (20.17)		89.76 (19.60)		0.98 (19.88)		–0.72 (13.77)		.72
	Noncompleters (n=209)		93.33 (25.09)		93.49 (24.83)		1.00 (12.89)		0.16 (8.64)		.79
**Slightly elevated (6.5<HbA_1c_<7.4%)**											
	All participants (N=296)		88.16 (22.36)		82.44 (19.37)		–5.14 (13.83)		–5.72 (12.61)		<.001
	Completers (n=182)		87.94 (20.60)		80.64 (16.87)		–7.27 (10.78)		–7.30 (11.34)		<.001
	Partial completers (n=42)		87.37 (24.09)		80.83 (18.78)		–4.66 (20.47)		–6.54 (15.17)		.008
	Noncompleters (n=72)		89.17 (25.67)		87.94 (24.27)		0.02 (14.79)		–1.23 (13.15)		.43
**Normal (HbA_1c_<6.5%)**											
	All participants (N=257)		85.67 (21.79)		81.27 (18.06)		–2.79 (19.70)		–4.41 (14.19)		<.001
	Completers (n=155)		84.93 (21.34)		78.00 (17.46)		–6.65 (13.70)		–6.93 (12.56)		<.001
	Partial completers (n=55)		85.76 (24.33)		85.79 (18.19)		5.58 (31.97)		0.03 (18.80)		.99
	Noncompleters (n=47)		88.04 (20.38)		86.77 (17.74)		0.14 (13.74)		–1.27 (11.02)		.43

## Discussion

The Low-Carb Program is a digitally delivered, peer-supported, nutrition-focused, structured 10-week health intervention aimed at improving glycemic control, reducing hypoglycemic medication use, and promoting weight loss among adults with type 2 diabetes. This was not a randomized controlled trial, so we cannot compare the 12-month results to a control or standard-of-care group; therefore, the results of our trial should be interpreted cautiously because the study used convenience sampling, open-label, single-arm design, pre-post self-reported outcomes, and 71% of participants reported outcomes at 12 months. Even so, when adults with type 2 diabetes participate in the Low-Carb Program, and especially when they finish all 10 modules of the program, they report significantly reduced HbA_1c_, weight loss, and reduced medications. The percentage of individuals with an HbA_1c_ level less than 6.5% (indicating good diabetes control) increased from 25.70% (257/1000) to 50.30% (503/1000). Furthermore, 46.00% (464/1000) of participants lost at least 5% of their body weight. Also, of participants who were taking at least one hypoglycemic diabetes medication at baseline, 289/714 (40.5%) reduced one or more of these medications.

The percentage of individuals with an HbA_1c_ level of less than 6.5% increased from 25.70% (257/1000) to 50.30% (503/1000). This degree of control, when achieved through pharmacotherapy, is often accompanied by weight gain and risk for hypoglycemic events [27]. Indeed, as the now famous Action to Control Cardiovascular Risk in Diabetes (ACCORD) study reported, intensive hypoglycemic medical therapy “increased mortality and did not significantly reduce major cardiovascular events” [28].

As in other studies using a carbohydrate-restricted dietary approach, including Dr Unwin’s in-person program on which the Low-Carb Program was partially modeled [14,17,29], we achieved HbA_1c_ reduction with weight loss and decreased hypoglycemic medication use. This approach is given further credence by a recent meta-analysis, which concluded that carbohydrate-reduced interventions improve glucose control, in addition to other positive health effects such as improved triglyceride and high-density lipoprotein cholesterol [30].

Our study has several limitations. Although we encouraged participants to eat a carbohydrate-restricted diet, we did not measure their dietary intake. We also measured health outcomes (weight, glycemic control, and medication changes) using self-report, rather than measuring them directly or through medical records. However, previous research has found that these self-reported health outcomes can be quite close to actual values [31,32]. Another limitation was our rate of delivering the entire intervention, as only 528 (52.8%) completed all modules. However, a high rate (70.8%) reported 12-month outcomes. On the other hand, given that this program was entirely automated and had a wide reach, a large number of individuals were able to complete the program.

For participants who fully engage, an automated online program teaching a carbohydrate-reduced diet to adults with type 2 diabetes may facilitate glycemic control, weight loss, and reduced need for hypoglycemic medication. Although our design does not support causal conclusions, the program may be a useful adjunct for lifestyle self-management for adults with type 2 diabetes.

## Abbreviations

HbA_1c_: glycated hemoglobin A_1c_
IRB: Institutional Review Board
